# Pneumococcal Antibody Concentrations of Subjects in Communities Fully or Partially Vaccinated with a Seven-Valent Pneumococcal Conjugate Vaccine

**DOI:** 10.1371/journal.pone.0042997

**Published:** 2012-08-14

**Authors:** Martin O. C. Ota, Anna Roca, Christian Bottomley, Philip C. Hill, Uzochukwu Egere, Brian Greenwood, Richard A. Adegbola

**Affiliations:** 1 Medical Research Council Unit, Banjul, The Gambia; 2 Barcelona Center for International Health Research (CRESIB), Institut d’Investigacions Biomediques August Pi i Sunyer, Universitat de Barcelona, Barcelona, Spain; 3 Centre for International Health, School of Medicine, University of Otago, Dunedin, New Zealand; 4 GlaxoSmithKline Biologicals, Wavre, Belgium; 5 MRC Tropical Epidemiology Group, London School of Hygiene and Tropical Medicine, London, United Kingdom; 6 Faculty of Infectious and Tropical Diseases, London School of Hygiene and Tropical Medicine, London, United Kingdom; Instituto Butantan, Brazil

## Abstract

**Background:**

A recent trial with PCV-7 in a rural Gambian community showed reduced vaccine-type pneumococcal carriage in fully vaccinated compared with control communities. We measured pneumococcal polysaccharide antibody concentrations in this trial to understand further the mechanisms underlying the observed changes.

**Methods:**

A single-blind, cluster-randomized (by village) trial was conducted in 21 Gambian villages. In 11 villages, all residents received PCV-7 (Vaccine group); in 10 control villages only children <30 months old or those born during the study received PCV-7. Subjects over the age of 30 months resident in vaccine villages received a single dose of PCV-7 whilst those in control villages received a single dose of a serogroup C meningococcal conjugate vaccine. Serum antibody concentrations against specific pneumococcal polysaccharides were measured in approximately 200 age-stratified subjects before, 4–6, 12 and 24 months following vaccination.

**Results:**

Baseline pneumococcal antibody concentrations were generally high and increased with age up to 10 years. One dose of PCV-7 increased geometric mean antibody concentrations (GMC) in vaccinated versus control villages for vaccine serotypes 6B and 18C, and 4 and 18C, in the young (under 5 years) and older age groups (5+ years) respectively. There were significantly higher proportions of subjects in the vaccinated than in the control communities with an antibody concentration believed to protect against carriage (>5.0 µg/mL) for all but serotype 9V of the PCV-7 serotypes in the older group, but not in the younger age group.

**Conclusion:**

Higher antibodies in vaccinated communities provide an explanation for the lower pneumococcal carriage rates in fully vaccinated compared to control communities.

**Trial Registration:**

Controlled-Trials.com ISRCTN51695599 51695599.

## Introduction

Pneumonia is one of the leading causes of mortality in children <5 years old. It is responsible for 1.6 million (18%) of the 8.8 million deaths annually in children in this age group [1], with 50% of these deaths occurring in sub-Saharan Africa [2]. *Streptococcus pneumoniae* (the pneumococcus) accounts for 30–50% of pneumonia-related deaths, and is a leading cause of death in children <2 years of age in developing countries [3], [4], [5]. In The Gambia, *S. pneumoniae* is a common cause of pneumonia, septicemia and meningitis [5], [6], [7], [8]. Population-based studies undertaken in Upper River Region, The Gambia showed an incidence rate of invasive pneumococcal disease (IPD) among infants approximately 10–20 times higher than that found in Caucasian populations in Europe and the United States of America [6], [9], [10]. High rates of IPD in developing countries are associated with high rates of nasopharyngeal carriage of pneumococci [11], [12].

**Table 1:** Characteristics of individuals sampled in cross-sectional surveys.

	BASELINE		CS1		CS2		CS3	
Age at vaccination	N	%	N	%	N	%	N	%
30 months–4 yrs	44	24	34	25	19	19	8	12
5 yrs–9 yrs	22	12	17	13	16	16	13	20
10–19 yrs	35	19	21	16	24	24	12	18
20–39 yrs	32	17	22	16	19	19	22	34
40 yrs+	38	20	40	30	21	21	10	15
Unknown	16	9	0	0	0	0	0	0
Total	187	100	134	100	99	100	65	100
**Sex**								
Female	89	48	65	49	59	60	23	35
Male	98	52	69	51	40	40	42	65
Total	187	100	134	100	99	100	65	100
**Group**								
Vaccinated	89	48	60	45	43	43	31	48
Not Vaccinated	98	52	74	55	56	57	34	52
Total	187	100	134	100	99	100	65	100
**Days post vaccination**								
**Median (IQR)**	0	(−77,0)	116	(99,127)	364	(281,365)	663	(626,713)

A summary of the number of subjects, age category, gender and groups evaluated at baseline and at each cross-sectional samplings (CSS1 to CSS3) are shown.

Vaccination provides an attractive and cost-effective intervention to prevent IPD. The introduction of a seven-valent pneumococcal conjugate vaccine (PCV-7) into routine immunization programs has significantly reduced the incidence of IPD in young children and adults in many countries [13]. It has also significantly reduced the carriage rate of vaccine serotypes in the nasopharynx, interrupting transmission [14], [15]. The protection afforded by pneumococcal conjugate vaccines is limited mainly to the serotypes contained within the vaccine [16], [17], and serotype replacement may occur [18], [19], [20].

To investigate the impact of community wide vaccination with PCV-7 on nasopharyngeal carriage of pneumococci, a cluster Randomized Clinical Trial (RCT) was conducted in a rural area of western Gambia in which one group of villages was fully-vaccinated (all residents) with PCV-7 (Vaccine group) while in other villages only children <30 months old and those born during the study period received PCV-7 (Control group) [21]. The trial showed an impressive reduction in nasopharyngeal carriage of pneumococci of vaccine type (VT) and a non-significant increase in the prevalence of pneumococci of non-vaccine type (NVT) in both study groups during the 22 months following PCV-7. This finding suggests that vaccination of young children had an indirect effect on nasopharyngeal carriage in adults by reducing transmission from children to adults. Vaccination of older children and adults provided limited added benefit. To investigate further the mechanisms underlying these findings we measured antibody concentrations to pneumococcal polysaccharide antigens of relevant serotypes in older children and adults from vaccinated and control groups before and at different time points after PCV-7 vaccination.

## Methods

### Study Site and Recruitment of Study Participants

Sera were obtained during the course of a single-blind, cluster-randomized (by village) trial conducted in 21 villages in the Sibanor district of the Western Region of The Gambia. Details of the study design and implementation have been reported previously [21]. Eleven villages were randomly assigned to one study group where all participants above the age of 30 months received PCV-7 and 10 to a second, control group, where all participants above the age of 30 months received a serogroup C meningococcal conjugate vaccine. Children less than 30 months of age received PCV-7 in all villages. The trial was conducted according to the principles of International Conference on Harmonisation - Good Clinical Practice guidelines, registered ISRCTN51695599, and approved by the Gambian Government/MRC Joint Ethics Committee and by the Ethics Committee of the London School of Hygiene and Tropical Medicine.

### Vaccines and Study Groups

PCV-7 (Prevnar®;Wyeth Lederle Pediatric Vaccines), containing 7 conjugated polysaccharides (4, 6B, 9V, 14, 18C, 19F and 23 F) was used for the appropriately randomized participants. A meningococcal polysaccharide C conjugate vaccine, also provided by Wyeth Lederle Pediatric Vaccines, was used in the control villages. This comprises purified short-chain oligosaccharides derived from serogroup C meningococcal capsular polysaccharide, coupled to CRM_197_, a nontoxic mutant diphtheria toxin, given with 0.5 mg of Al_2_PO_4_ adjuvant.

**Figure 1:**
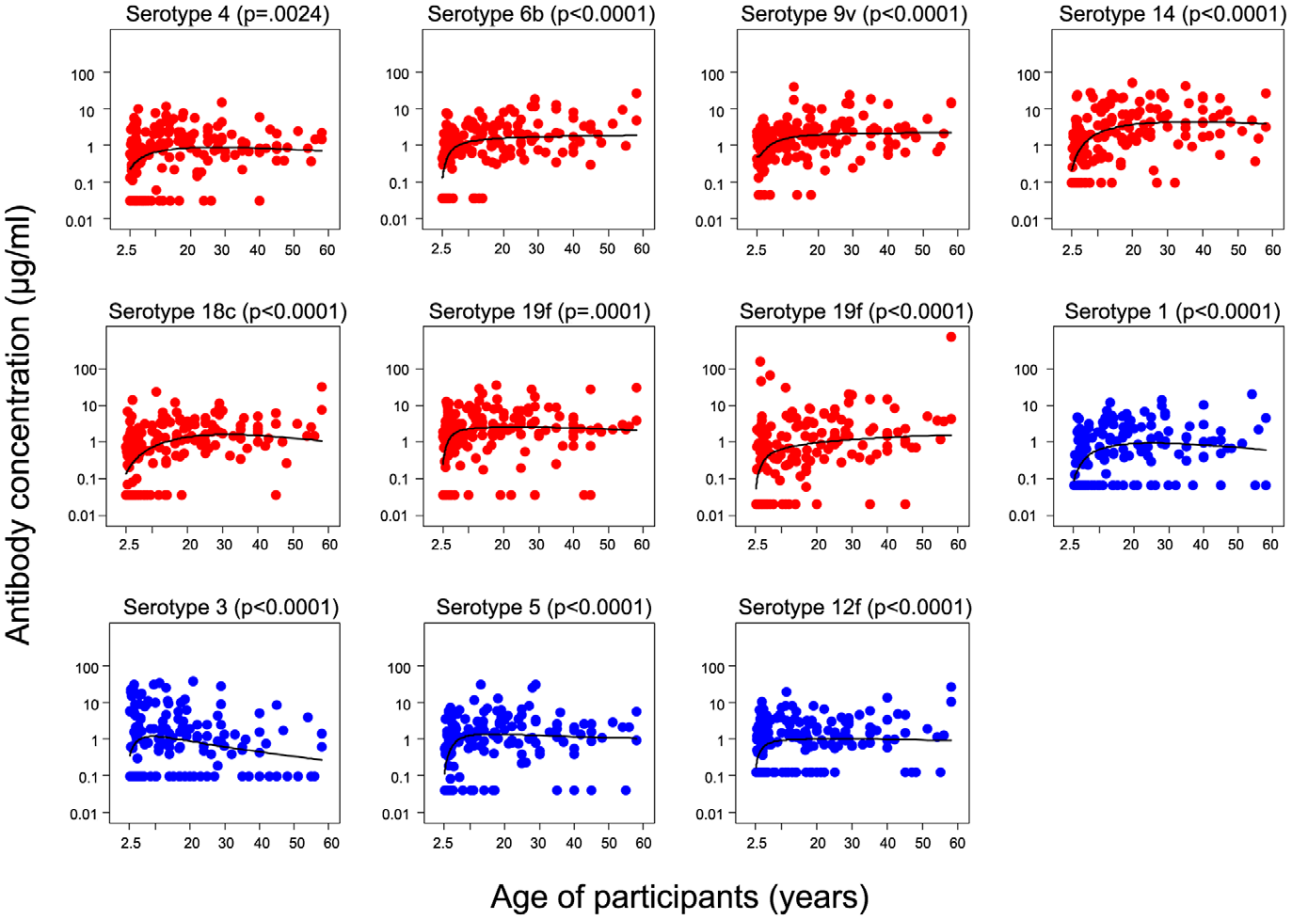
Variation of anti-capsular pneumococcal antibody concentrations by age at baseline. The distribution of baseline anti-pneumococcal capsular IgG antibody concentrations for PCV-7 and non-PCV-7 (1, 3, 5 and 12F) seroypes according to the age of subjects is shown. Data from Vaccinated and Control villages are combined for this baseline analysis. P-values are for the association between IgG and age.

**Table 2:** Geometric mean IgG pneumococcal antibody concentrations to individual polysaccharides at baseline and the proportion of participants with concentrations above threshold concentrations.

	30 months – 4 years						5 years+					
	Control Villages			Vaccinated villages			Control Villages			Vaccinated villages		
Serotypes	GM	% ≥0.35µg/ml	% >5.0µg/ml	GM	% ≥0.35µg/ml	% >5.0µg/ml	GM	% ≥0.35µg/ml	% >5.0µg/ml	GM	% ≥0.35µg/ml	% >5.0µg/ml
**PCV-7**												
4	0.42	51.7	3.3	0.24	50.9	2.8	0.5	75.8	5.8	0.73	78.3	11.2
6B	0.36	60	3.3	0.3	63	11.1	0.95	79.5	12.7	1.53	96.1	12.8
9V	0.54	65	0	0.84	71.3	11.1	1.42	92	6	2.14	96	23
14	0.51	53.3	5	0.85	70.4	11.1	2.27	80.5	38.2	2.26	93.6	29.7
18C	0.21	45	3.3	0.26	49.1	14.8	0.62	73.2	1.7	1.13	85.9	13.6
19F	0.87	75	21.7	1.2	85.2	25	2.24	91.2	23.7	2.47	94.4	29.6
23F	0.15	40	3.3	0.26	50	13.9	0.6	73.2	11.3	0.88	73.7	16.3
**Non-PCV-7**												
1	0.18	28.3	0	0.22	35.2	0	0.6	70.2	5.5	0.79	72.3	16.9
3	0.71	51.7	25	0.58	54.6	18.5	0.63	65	13.5	0.49	49.2	10.2
5	0.37	58.3	6.7	0.4	61.1	11.1	0.95	87.7	7.8	1.73	94.5	18.3
12F	0.31	38.3	0	0.43	43.5	6.5	0.64	65	6.7	1.13	78.1	12.1

At baseline the vaccinated and control communities were combined, and analysed according to those between 30 months and 4 years of age and those 5 years and above. The geometric means (GM) of the antibody concentrations, the proportions with concentrations that are postulated to protect against invasive pneumococcal disease (≥0.35 mg/ml) and nasopharyngeal carriage (≥5.0 µg/ml) are shown.

All study children between 2 and 30 months of age received PCV-7 according to the following schedule: 3 doses at 2, 3 and 4 months of age for infants born during the period of study, 3 doses at monthly intervals for those aged between 2 and 11 months of age at the time of the mass vaccination campaign, and 2 doses at monthly intervals for those aged between 12 and 30 months at that time. Subjects above the age of 30 months received either one dose of PCV-7 if resident in the vaccine group of villages or one dose of meningococcal C conjugate vaccine if resident in the control group of villages. All vaccines were given by deep intramuscular injection.

**Figure 2:**
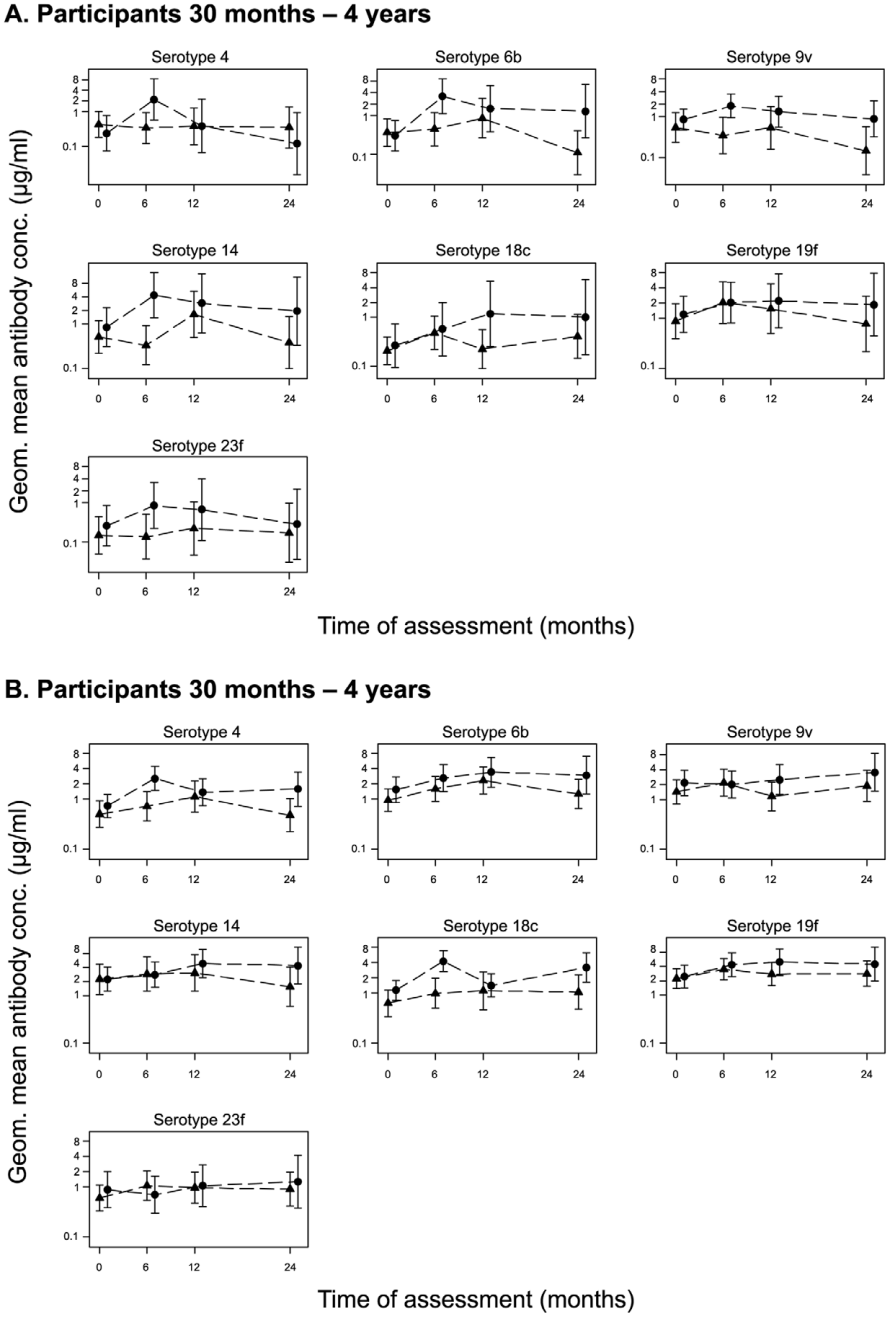
Geometric mean (95% CI) IgG response to vaccine serotypes in vaccinated and control communities. The GM of antibody concentrations to the PCV-7 serotypes are plotted at the time of CSS 1 to 3 coinciding with the three different time points after vaccination, 4–6 months, 12 months and 24 months for the vaccinated (circles) and control (triangles) communities for participants 30 months to 4 years (A), and 5 years and above (B). Note that samples were taken at the same periods but shifted in figure to avoid overlaps that might hide some points.

### Cross-sectional Surveys and Sampling Methods

Samples for serological assays were collected shortly before vaccination (between 1 day and 1 week) and at three different time points after vaccination (4–6 months, 12 months and 24 months). Sera were collected from approximately 200 subjects randomly selected within three age strata (50 aged 30 months –4 years, 50 aged 5–15 years and 100 aged >15 years) during each of these surveys. A separate random selection of individuals was carried out at each survey. Two ml of venous blood was collected for the measurement of anti-capsular pneumococcal antibodies. A mass campaign by the Gambian National Trachoma Elimination Programme which involved administration of one dose of azithromycin to all individuals older than 6 months of age, except pregnant women, started at the time of the last Cross Sectional Survey (CSS) in most study villages, but this did not change the blood sampling schedule.

**Table 3:** Geometric mean pneumococcal polysaccharide antibody concentrations in vaccinated and control villages following vaccination with 1 dose of PCV-7 or serogroup C meningococcal conjugate vaccine (data from CSS1–CSS3 combined).

	30 months –4 years					5 years+					
Serotypes	Control	Vacc	Ratio	Ratio adj†	P value	Control	Vacc	Ratio	Ratio adj†	P value	P value int‡
**PCV-7**											
4	0.42	0.60	1.44	1.46(0.19,11.34)	0.694	0.77	1.99	2.58	2.53(1.04,6.20)	0.042	0.431
6B	0.61	2.28	3.72	3.73(1.09,12.85)	0.038	1.76	2.91	1.66	1.66(0.68,4.03)	0.247	0.190
9V	0.54	1.38	2.55	2.20(0.77,6.29)	0.130	1.97	2.53	1.29	1.09(0.47,2.52)	0.836	0.173
14	0.67	3.73	5.58	3.98(0.98,16.21)	0.053	2.69	4.64	1.72	1.72(0.64,4.66)	0.264	0.022
18C	0.39	0.70	1.77	2.70(1.14,6.37)	0.027	1.04	2.80	2.70	2.63(1.11,6.22)	0.030	0.305
19F	2.14	2.33	1.09	1.36(0.57,3.26)	0.463	3.58	5.24	1.46	1.46(0.78,2.72)	0.220	0.816
23F	0.24	0.8	3.40	2.33(0.57,9.54)	0.219	1.01	0.70	0.69	0.67(0.22,2.04)	0.458	0.080
**Non-PCV-7**											
1	0.48	0.35	0.72	0.64(0.20,2.00)	0.408	1.01	0.99	0.98	0.93(0.45,1.90)	0.825	0.767
3	0.45	0.74	1.62	1.51(0.25,8.97)	0.626	0.47	0.72	1.53	1.57(0.68,3.62)	0.268	0.749
5	1.40	0.88	0.62	0.62(0.21,1.88)	0.370	2.06	1.85	0.9	0.99(0.56,1.75)	0.958	0.248
12F	0.73	0.44	0.61	0.53(0.17,1.68)	0.255	1.19	0.97	0.81	0.80(0.36,1.78)	0.570	0.253

Due to similar kinetics in response to a single dose of PCV-7 at the three times points of assessment, data from CSS1 to CSS3 were combined to increase study power. The ratio of the GM in the vaccinated and control communities are derived and adjusted for the baseline values (†).

‡ indicate the p-values for test for a difference in the effect of vaccine in the two age groups (interaction).

### Measurement of Antibody Concentration to Pneumococcal Serotype Polysaccharides

Venous blood was allowed to clot at room temperature and serum separated and stored at −70°C until used for the measurement of anti-pneumococcal antibody concentrations. Test samples and controls were assayed using an adapted WHO ELISA protocol [17] for type-specific IgG antibodies to the pneumococcal polysaccharides of PCV-7 serotypes (4, 6B, 9V, 14, 18C, 19F, and 23F) and to four other serotypes of local interest (1, 3, 5, 12F). Serotypes 1 and 5 are highly prevalent among IPD cases in the Gambia and many other parts of the developing world but rarely found in carriers. Serotype 3 is highly prevalent in carriers but found less frequently in cases of IPD. Serotype 12 is rarely found in either carriers or cases of IPD [6], [11], [22]. Laboratory assays were done blinded to the participant’s study group.

**Figure 3:**
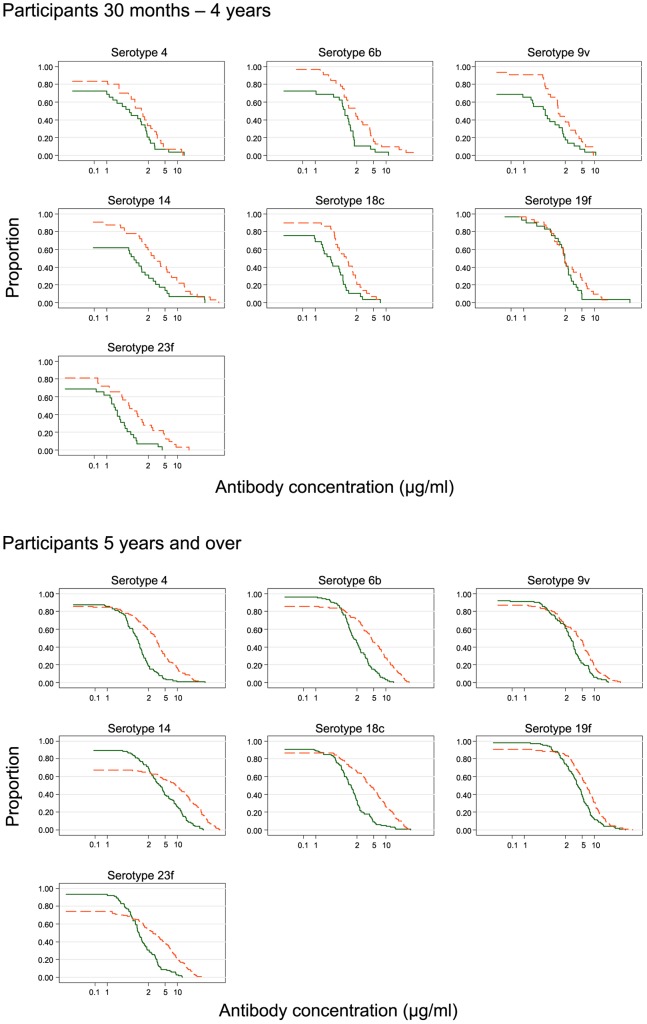
Reversed cumulative plots showing the proportion of individuals with an IgG pneumococcal antibody concentration above a particular value following vaccination. The proportion of individuals with serotype-specific IgG antibody concentrations from vaccinated (broken lines) and control (solid lines) communities in the (A) young and (B) older groups using combined data from CSS1–CSS3 are shown.

**Table 4:** Proportion of subjects with pneumococcal polysaccharide antibody concentrations >5.0 µg/mL in vaccinated and control villages following vaccination with 1 dose of PCV-7 or serogroup C meningococcal conjugate vaccine (data from CS1–CS3 combined).

	30 months – 4 yrs					5 years+					
Serotypes	Control	Vacc	Difference	Difference adj†	P value	Control	Vacc	Difference	Difference adj†	P value	P value int‡
**PCV-7**											
4	0.06	0.14	0.08	−0.02(−0.18,0.14)	0.787	0.05	0.30	0.25	0.25(0.04,0.47)	0.024	0.160
6B	0.06	0.20	0.14	0.05(−0.16,0.25)	0.619	0.13	0.49	0.37	0.36(0.19,0.54)	<0.001	0.224
9V	0.06	0.10	0.05	0.07(−0.11,0.26)	0.397	0.17	0.45	0.28	0.19(−0.08,0.46)	0.149	0.009
14	0.18	0.48	0.30	0.15(−0.14,0.44)	0.295	0.36	0.63	0.27	0.26(0.03,0.49)	0.031	0.524
18C	0.03	0.04	0.01	0.03(−0.08,0.13)	0.600	0.09	0.53	0.44	0.38(0.15,0.60)	0.002	0.004
19F	0.14	0.29	0.15	0.18(−0.23,0.59)	0.366	0.34	0.69	0.35	0.35(0.14,0.56)	0.003	0.442
23F	0.00	0.18	0.18	0.09(−0.03,0.21)	0.120	0.07	0.35	0.28	0.28(0.12,0.45)	0.002	0.113
**Non-PCV-7**											
1	0.19	0.15	−0.04	−0.14(−0.42,0.15)	0.311	0.10	0.18	0.08	−0.01(−0.22,0.21)	0.940	0.795
3	0.12	0.29	0.17	0.12(−0.15,0.39)	0.369	0.11	0.14	0.03	0.03(−0.11,0.18)	0.640	0.269
5	0.10	0.01	−0.09	−0.03(−0.12,0.05)	0.42	0.15	0.23	0.07	0.12(0.01,0.24)	0.042	0.048
12F	0.08	0.01	−0.07	−0.06(−0.23,0.11)	0.472	0.07	0.09	0.02	0.02(−0.07,0.11)	0.623	0.098

The difference in the proportions in the vaccinated and control communities that achieved anti-pneumococcal antibody concentrations ≥5.0 µg/ml were compared in the younger (30 months to 4 years) and older (5 years +) age groups.

† adjusted for baseline prevalence,

‡ p-value for interaction (i.e., to test for a difference in the effect of vaccine in the two age groups).

### Statistical Analyses

All children who had been vaccinated at ages 2–30 months were excluded from the serological surveys since these children would have received PCV-7 in both sets of villages and would not have provided information on the differential effect of community wide vaccination with PCV-7. Analysis is based on village level summaries of the data calculated at baseline, and at three post vaccination surveys. Data from the three cross-sectional surveys conducted after vaccination were combined to improve statistical power when looking at geometric means and percentage of responders by individual serotype. The distribution of IgG responses post vaccination was displayed graphically by plotting the survival function. We used ratios of geometric mean IgG and differences in the proportion of subjects with an IgG antibody concentration >0.35 µg/ml and >5 µg/ml [23], concentrations believed to provide protection against IPD and carriage respectively, to compare vaccinated and control villages. The ratio of geometric means was calculated by back-transforming (i.e., taking the anti-log) of the difference in mean log IgG between vaccinated and control villages. In the absence of a recorded IgG response, a value half the lower limit of detection was assumed. Analysis of covariance (ANCOVA) was used to adjust for baseline levels of the serotypes in the villages in all analyses. P-values for interactions between age and trial arm were obtained using the method described by Cheung et al [24].

Linear regression models were fitted to combined baseline data from both PCV-7 and non-PCV-7 villages to explore the relationship between log IgG response and age. The relationship was modelled using fractional polynomials of degree 2. Robust variance estimates were used to allow for heterogeneous variance, and the Wald test was used to assess statistical significance.

## Results

### Study Profile

Six hundred and sixty-two blood samples were collected during the trial; 485 (73%) (187 in the baseline CSS and 298 in the post-vaccination CSS) were processed and included in the analysis. The rest were lost due to storage problems. The characteristics of the individuals whose samples were lost were not different from those included in the analysis (Table S1). The majority of the lost samples were collected during the last CSS. Table 1 shows mean age and sex of the individuals with samples available for analysis according to CSS. The number and age of subjects sampled was similar at each survey.

### Anti-pneumococcal Antibodies in Communities Before PCV-7

The concentration of antibodies to several pneumococcal polysaccharides prior to vaccination by age is shown in Fig. 1. Concentrations of antibodies increased with age, reaching a plateau at about ten years for most serotypes, but for serotype 3 there was a decline following an initial increase. As children under the age of 5 years are a potential target for ‘catch-up’ vaccination programmes we have evaluated antibody responses in children aged 30 months –4 years at vaccination and in older subjects (5 years and older) separately. The GM antibody concentrations at baseline were lower in the younger age group for all except serotype 3, and consequently the proportions >0.35 µg/ml were also lower compared to the older group (Table 2). The antibody concentrations in both the young and older groups were highest for serotype 19F, proportions with concentrations >5.0 µg/ml were low in both age groups.

### Antibody Concentrations after a Single Dose of PCV-7

The kinetics of the antibody response following one dose of PCV-7 in vaccinated communities are shown in Figure 2. Antibody concentrations post vaccination were consistently higher in vaccinated than in control communities for serotypes included in PCV-7. Geometric mean IgG concentrations at CSS1 (first post vaccination survey) were significantly higher (p<0.05) among PCV7 than control villages for serotypes 6B (adjusted OR = 10.89; 95% CI: 1.06,112.32; p = 0.05) and 14 (adjusted OR = 10.73; 95% CI: 1.51,76.27, p = 0.02) in the younger age group and for serotypes 14 (adjusted OR = 3.50; 95% CI: 1.51,8.07; p = 0.01) and 18C (adjusted OR = 4.77; 95% CI: (1.68,13.50), p = 0.01) in the older age group. Twelve months after vaccination (CSS2), the difference between arms was significant for serotype 18C in the younger age group (adjusted OR = 6.71; 95% CI: 1.06,42.63; p = 0.05). Twenty four months after vaccination (CSS3), differences between arms were significant for serotypes 6B (adjusted OR = 8.33, 95% CI: 2.88,24.05, p = 0.005) and 9V (adjusted OR = 8.16; 95% CI: 3.27,20.38; p = 0.003) in the younger age group and for serotypes 9V (adjusted OR = 2.04; 95% CI: 1.09,3.79; p = 0.03) and 14 in the older age group (adjusted OR = 2.87; 95% CI:1.27,6.46; p = 0.02).

Data from the post vaccination surveys (CSS1 to CSS3) combined (Table 3), showed that the GM antibody concentrations were higher in the vaccinated than in the control villages for all vaccine serotypes in the younger age group, reaching statistical significance for serotypes 6B and 18C. In the older age group, the GM antibody concentrations were significantly higher in the vaccinated compared with the control group for serotypes 4 and 18C (Table 3). There was a significant difference in the effect of the vaccine between the young and older groups for serotype 14 (ratios of 3.98 vs. 1.72, p = 0.022) (Table 3). As expected there was no difference in GM ratios between the vaccine and control groups for the non-PCV-7 serotypes that were measured (1, 3, 5, and 12F). GM ratios were not significantly different in males and females (data not shown).

We examined the distribution of serotype-specific IgG antibody concentrations from vaccinated and control communities in the young and older groups using combined data from CSS1–CSS3 (Figures 3A & B). The curves cross in the older age group as the positive effect of vaccination only became apparent at higher IgG antibody concentrations (Figure 3B). The proportion of subjects who had antibody concentrations >5.0 µg/ml to PCV-7 serotype polysaccharides in the vaccinated villages ranged from 4–48% in the younger age group (serotypes 18C and 14, respectively) and from 30–69% in their older counterparts (serotypes 4 and 19F, respectively). The proportion of subjects who had an antibody concentration >5 µg/ml to PCV-7 serotypes was consistently higher in the vaccinated than in the control villages (Table 4) with absolute differences ranging from 1–30% (serotypes18C and 14, respectively) among the younger group, and from 25–44% (serotypes 4 and 18C, respectively) for the older group. The differences in the prevalence of antibodies >5.0 µg/ml between subjects from the vaccinated and control villages were not statistically significant in the younger age group but the differences were significant in the older age group for all PCV-7 serotypes except 9V (Table 4). Surprisingly, a significantly higher proportion of subjects in the vaccinated community had antibodies >5.0 for serotype 5 that is not contained in PCV-7. The effect of vaccination (as measured by the absolute difference in the proportion with antibody concentrations >5.0 µg/ml between control and vaccinated villages) was different in the two age categories for serotypes 9V and 18C, implying significant interaction between age and the immune response to different polysaccharides (Table 4).

## Discussion

We have evaluated antibody profiles in a group of communities in which everybody received PCV-7 and compared these to another group of control communities in which only children younger than 30 months of age received PCV-7. As anticipated, the baseline antibody concentrations were similar in the two groups, but after 1 dose of PCV-7, subjects in the vaccinated group had significantly increased antibody concentrations compared with subjects in control communities and a greater proportion of vaccinated subjects had antibody concentrations that are thought to protect against carriage. Younger participants had lower antibody concentrations than older subjects at baseline, as expected, but their responses to PCV-7 as determined by the ratio of GM to the control group tended to be relatively greater than that of their older counterparts.

Prior to vaccination, a high proportion of participants had antibody concentrations ≥0.35 µg/mL, a concentration that has been considered to be protective against IPD. Similar results have been observed in another Gambian community [17], as well as in other developing countries [25]. This is most likely a result of responses to nasopharyngeal carriage of pneumococci of these serotypes, which is high in this population [11], and similar settings [26]. The prevalence of individuals with protective antibody concentrations against serotypes found infrequently in the community (such as serotype 12F) was also high, although lower than for common VT serotypes. Baseline concentrations of antibody increased with age, reaching a plateau at about the age of 10 years suggesting an impact due to repeated colonisation.

For the majority of vaccine serotypes, one dose of PCV-7 significantly increased the proportion of subjects who had antibody concentrations at the level presumed to protect against carriage. Such a response to a single dose of PCV-7 is in keeping with a typical anamnestic response of the immune system that has been primed, in this case most likely by natural exposure to the pneumococcus through carriage. The increase of individuals with antibody concentrations associated with protection against carriage in the vaccinated group is in agreement with the finding that although the incidence of VT carriage fell in older children and adults in both study groups during the observation period it was more marked in the vaccination group [21]. Surprisingly, the positive effect of vaccination became obvious only at higher IgG concentrations in the older group. The mechanism behind this is not clear, but could be related to a relatively higher proportion having existing antibodies before vaccination or it could be a chance finding. There was a significantly higher proportion of subjects in the vaccinated community that had antibodies >5.0 for serotype 5 that is not contained in PCV-7. We do not understand the reason behind this observation. To the best of our knowledge there is no reported serotype 5 cross-reacting antigen associated with PCV-7 that could be responsible for this. It may be that the increased prevalence of serotype 5 carriage in the vaccinated villages [21] might have induced antibodies that we have measured. The relative increase in antibody GM ratios in the vaccinated compared to the control group was more pronounced in the younger participants, who had lower prevaccination antibody concentrations as compared to their older counterparts. The induction of a strong and sustained antibody response with a single dose of PCV-7 in infants has been described previously [27], and could support the WHO recommendation of a catch-up immunization with one dose of PCV for children aged 12–24 months at the point of introducing pneumococcal conjugate vaccines into the expanded programme on immunization (EPI) [28]. Moreover, in developing countries where the burden of IPD is high below 3 months of age [29], our data support the idea that a single dose of PCV administered to pregnant mothers could enhance the concentrations of pneumococcal antibodies passed on to protect against IPD in early infancy before infant doses of PCV are administered [30], [31].

Our study faced a number of challenges. First, some of the samples collected for the study could not be used for the analysis as they had suffered storage problems. However, the characteristics of individuals from whom the samples were processed and those whose samples had to be discarded were similar so this should not have introduced bias (Table S1). As the final number of samples processed is slightly lower than initially planned, the power to detect differences between age groups was compromised, especially in the last CSS. Furthermore, the last post-vaccination survey was disrupted by the administration of azithromycin to individuals in several study villages as part of a national trachoma elimination program that had not been envisaged when the trial was planned (treatment given between 1 day and 2 weeks before the samples were collected). However, we do not anticipate that this treatment would have had any immediate effect on antibody concentrations. We note that in addition to antibody concentrations in serum measured by ELISA, antibody function, as measured by opsonophagocytic activity and avidity, plays an important role in protection against pneumococcal infection [32] and these functions were not measured in our study.

This study has shown high baseline anti-capsular pneumococcal antibody concentrations in rural Gambian villages which have high rates of pneumococcal carriage. Antibody concentrations and proportions of individuals with concentrations protective against carriage were higher in vaccinated than control villages and this is likely to have contributed to the difference in carriage rates seen between the two groups of villages [21]. Vaccination of children aged 30 months –4 years with a single dose of PCV-7 increased antibody concentrations to most VT polysaccharides and might be a useful strategy in communities where high risk of pneumococcal disease in children extends beyond the first two years of life.

## Supporting Information

Table S1 **Comparison of the characteristics of individuals whose samples were analysed to those lost.**
(DOCX)
