# Supplementary material for: Pneumococcal Antibody Concentrations of Subjects in Communities Fully or Partially Vaccinated with a Seven-Valent Pneumococcal Conjugate Vaccine
Source: PLoS One. 2012 Aug 14;7(8):e42997. doi: 10.1371/journal.pone.0042997 (PMC3419246; doi:10.1371/journal.pone.0042997)
Supplement: Table S1 — Comparison of the characteristics of individuals whose samples were analysed to those lost. (DOCX) [file pone.0042997.s001.docx]

**Supplementary Table 1. Characteristics of individuals whose samples were analysed and those whose samples were lost.**

|  | In Study | | Lost |  | P-value |
| --- | --- | --- | --- | --- | --- |
|  | No. | % | No. | % |  |
| **Cross-sectional survey** |  |  |  |  |  |
| Baseline | 187 | 38.6 | 13 | 7.3 |  |
| CS1 | 134 | 27.6 | 42 | 23.7 |  |
| CS2 | 99 | 20.4 | 48 | 27.1 |  |
| CS3 | 65 | 13.4 | 74 | 41.8 |  |
| Total | 485 | 100 | 177 | 100 | <0.001 |
| **Age** |  |  |  |  |  |
| 30mnths-5yrs | 116 | 23.9 | 41 | 23.2 |  |
| 6yrs-9yrs | 52 | 10.7 | 25 | 14.1 |  |
| 10-19yrs | 100 | 20.6 | 42 | 23.7 |  |
| 20-39yrs | 93 | 19.2 | 37 | 20.9 |  |
| 40yrs+ | 124 | 25.6 | 32 | 18.1 |  |
| Total | 485 | 100 | 177 | 100 | 0.268 |
| **Gender** |  |  |  |  |  |
| Female | 236 | 48.7 | 98 | 55.4 |  |
| Male | 249 | 51.3 | 79 | 44.6 |  |
| Total | 485 | 100 | 177 | 100 | 0.127 |
| **Group** |  |  |  |  |  |
| Control | 223 | 46 | 95 | 53.7 |  |
| Vaccinated | 262 | 54 | 82 | 46.3 |  |
| Total | 485 | 100 | 177 | 100 | 0.08 |
